# Elucidation of chromatographic peak shifts in complex samples using a chemometrical approach

**DOI:** 10.1007/s00216-018-1173-9

**Published:** 2018-06-14

**Authors:** Pedro F. M. Sousa, Angela de Waard, K. Magnus Åberg

**Affiliations:** Unit for Analytical Chemistry, Department of Environmental and Analytical Chemistry, Stockholm University, SE-106 91 Stockholm, Sweden

**Keywords:** Chromatography, Alignment, PCA

## Abstract

Chromatographic retention time peak shifts between consecutive analyses is a well-known fact yet not fully understood. Algorithms have been developed to align peaks between runs, but with no specific studies considering the causes of peak shifts. Here, designed experiments reveal chromatographic shift patterns for a complex peptide mixture that are attributable to the temperature and pH of the mobile phase. These results demonstrate that peak shifts are highly structured and are to a high degree explained by underlying differences in physico-chemical parameters of the chromatographic system and also provide experimental support for the alignment algorithm called the generalized fuzzy Hough transform which exploits this fact. It can be expected that the development of alignment algorithms enters a new phase resulting in increasingly accurate alignment by considering the latent structure of the peak shifts.

**Electronic supplementary material:**

The online version of this article (10.1007/s00216-018-1173-9) contains supplementary material, which is available to authorized users.

## Introduction

Chromatographic retention mechanisms have been studied and modeled since the 1970s. In the field of quantitative structure–retention relationships (QSRR), the focus has been on predicting parameters, such as log *k*-values, log *P* values, log *D*-values, and retention factor ratios, which are based on molecular descriptors [1–4]. Other studies have targeted the effect of pressure on chromatographic selectivity for comparing the relatively new ultra-high-pressure liquid chromatography (UHPLC) to more traditional high-performance liquid chromatography (HPLC) separations operated at lower pressures [5, 6]. However, these approaches cannot be used to investigate the fine-structure of peak shifts arising under nominally identical chromatographic conditions.

Peak shifting is a common phenomenon observed in chromatographic separations that may cause problems identifying which peak is which, especially for datasets with a large number of samples and many peaks per sample. Failure to find the correct peaks that correspond to the same analyte between samples often results in poor statistical analysis. This problem has been coined the correspondence problem [7–12]. The causes of peak shifting in chromatography have been addressed and described in literature [12]. However, so far, the research focused on solving the correspondence problem has only aimed to make sure the data are properly aligned for statistical analysis, and several retention time alignment algorithms have been reported in literature, such as nearest-neighbor clustering [13, 14], binning [15, 16], and warping [17–20]. Some of these algorithms have been implemented in chromatography data analysis software, such as msInspect, MZmine, OpenMS, XCMS, or TracMass 2 [10, 21].

This work fuses alignment methodology with retention time modeling to improve the understanding of chromatographic retention in complex systems. For this purpose, a liquid chromatography coupled with mass spectrometry (LC/MS) method for analyzing a complex mixture was setup using an experimental design, with temperature of the column and pH of the mobile phase as variables provoking retention time shifts. The model sample used was a tryptic digest of human serum albumin (HSA), which is a mixture of peptides, resulting from the selective digestion by the enzyme trypsin. This enzyme cleaves proteins exclusively at specific amino acids along the sequence (C-terminal to arginine or lysine). Along with the theoretical peptides, other species may occur in the mixture, such as possible miscleavages and post-translational modifications in the protein. This moderate sample complexity provides a good model for this study, as the peptide elution is well distributed along the chromatogram and the experimental design conditions tested, i.e., pH of the mobile phase and column temperature, influence the retention times with different patterns depending on the peptide. In addition, we also want to demonstrate experimental evidence in favor of generalized fuzzy Hough transform (GFHT) alignment algorithm.

## Generalized fuzzy Hough transform

Peak alignment methods such as binning, nearest-neighbor, clustering, warping, or combinations of these may result in ambiguities in peak matching, especially when aligning peaks from very complex mixtures [22]. Recent development of alignment algorithms employing the generalized fuzzy Hough transform (GFHT) has been reported [8]. GFHT is a derivation of the Hough transform, initially applied in image analysis for the detection of patterns, such as lines or features in images. Recently, it was adapted and evolved into the GFHT with the purpose of aligning 1D NMR peak data [8]. More recently, GFHT has also been applied in the alignment of chromatographic peaks, as an additional alignment step in TracMass 2, an open-source program designed to align LC-MS (liquid chromatography coupled with mass spectrometry) data. An important feature that arises with GHFT is that the ambiguities in peak matching that frequently occur when using other alignment methods can be resolved. This is accomplished by pre-calibrating the model on the shifts of peaks with known correspondence by means of principal component regression (PCR). Then, the retention times of the peaks with unknown correspondence can be predicted [8–10]. This method assumes that peaks shift according to patterns that are attributed to causes related to chromatographic instability. These patterns, however, have not yet been attributed to specific causes and reported in literature. In this work, the column temperature and the pH of the mobile phase were taken as two of the several possible factors that may influence retention time shifts. Then, retention time shifts were modeled using these parameters in a controllable fashion, i.e., at predetermined levels using experimental design and in magnitudes that influence the retention times more than than expected random variations of these factors and other uncontrollable factors.

## Experimental design by MLR and PCA

Factorial design is a chemometrical tool designed to assess hypothetical contributions of factors towards a response of interest. The mathematical approach employed in factorial design is typically a multiple linear regression (MLR). A response data matrix **X** or vector **x** containing the results of the experiments is obtained according to a design of experiments and is regressed on a design matrix **D**. This matrix contains coded values corresponding to the levels of the factors studied and their interactions. The number of levels can vary although for simplicity the most common factorial designs have two levels [23].

1 $$ {\mathbf{X }}= {\mathbf{DB}} $$

The MLR coefficients **B**, which reflect the impact of the studied factors on the response(s), are obtained applying the pseudo-inverse,

2 $$ {\hat{\mathbf{B} }}= ({\mathbf{D}}^{\mathrm{T}} {\mathbf{D}})^{-1}{\mathbf{D}}^{\mathrm{T}} {\mathbf{X}} $$

Principal component analysis (PCA) decomposes a data matrix into abstract features and is represented algebraically as

3 $$ {\mathbf{X}} = {\mathbf{TP}}^{\mathrm{T}} + {\mathbf{E}} $$

where **X** is a data matrix, e.g., containing experimental responses, decomposed into scores **T** and loadings **P**. Scores contain structure as the relation between samples and size, loadings contain structure in the form of relations between variables, and **E** is the unexplained residual information of the data. For this work, an algebraic relation between MLR and PCA is established in order obtain the experimental design coefficients **B** from the response data **X** and the design matrix **D** via both methods (MLR and PCA). The experimental design matrix **D** and the regression coefficients **B**, analogously to PCA, contain structure related to size and to their relations between variables respectively. Therefore, **D** can be related to the scores **T** and **B** to the loadings **P**. A least squares conversion factor **C** is introduced to establish this relationship, where **D** **=** **TC** **+** *E*_*C*_. Because the scores matrix is not necessarily square, depending on the number of samples and rank, this equation can be solved applying the pseudo-inverse,

4 $$ {\hat{\mathbf{C}}} = ({\mathbf{T}}^{\mathrm{T}} {\mathbf{T}})^{-1} {\mathbf{T}}^{\mathrm{T}} {\mathbf{D}} $$

This conversion factor ${\hat {\mathbf {C}}}$ can convert scores into the design matrix or the design matrix into scores. Introducing ${\mathbf {D}} = {\mathbf {T}}{\hat {\mathbf {C}}}$ in the MLR equation results in ${\mathbf {X}} = {\mathbf {T}\hat {\mathbf {C]}}\mathbf {B}} + {\mathbf {E}}$, and relating to the PCA equation, ${\mathbf {P}}^{\mathrm {T}} \approx {\hat {\mathbf {C}B}}$. Therefore, the experimental design coefficients can be calculated from the PCA loadings and the conversion matrix as ${\mathbf {B}} = {\hat {\mathbf {C}}}^{-1}{\mathbf {P}}^{\mathrm {T}}$. If the number of principal components differs from the number of MLR parameters, the pseudo-inverse is used instead,

5 $$ {\hat{\mathbf{B}}}_{\text{PCA}} = ({\hat{\mathbf{C}}}^{\mathrm{T}} {\hat{\mathbf{C}}})^{-1} {\hat{\mathbf{C}}}^{\mathrm{T}} {\mathbf{P}}^{\mathrm{T}} $$

The analysis of experimental design factors from complex experimental design data can be assessed with ANOVA-simultaneous component analysis (ASCA). This algorithm can manage analyses of complex multivariate data that may contain underlying experimental design factors. ASCA is generalization ANOVA to the multivariate case by means of PCA. In this work, we have used ASCA and PCA together to analyze the studied factors and calculate the design of experiments (DoE) regression coefficients [24].

## Experimental

### Reagents, materials, and instrumentation

Human serum albumin (HSA), trypsin from bovine pancreas, dithiothreitol, iodoacetamide, ammonium bicarbonate, formic acid, and ammonium formate were purchased from Sigma-Aldrich (Steinheim, Germany). Gradient grade acetonitrile and analytical grade water were purchased from Honeywell Riedel-de Haën (Seelze, Germany). A HPLC-MS system composed of a Thermo Fisher Scientific (Waltham, MA) Q Exective HF orbitrap mass analyzer coupled with a Thermo Fisher Scientific (Waltham, MA) UltiMate BioRS HPLC, equipped with a Thermo Fisher Scientific (Waltham, MA) HyPURITY column (C–18, i.d. 2.1 mm, length 100 mm, particle size 3 *μ* m) was used in this study. The mass detector operated in full-scan positive mode, for *m*/*z* values ranging from 100 to 2000. The HPLC was programmed to run with a flow rate of 0.25 mL min^− 1^. The organic mobile-phase gradient was set to start at 5%, raising to 45% for 30 min, then to 100% for 10 min, and back to 5% for 5 min. The temperature of the column was set in each run according to the experimental design as described bellow.

### Sample preparation (HSA tryptic digest)

To 5 mL of human serum albumin (150 *μ* M in 100 mM ammonium bicarbonate), 5 mL of dithiothreitol (100 mM) were added and let to react for 30 min at 60 ^∘^C. After cooling down, 5 mL of iodoacetamide (100 mM) was added and the mixture was kept in the dark for 30 min at room temperature. Five milliliters of trypsin (150 *μ* M in water) was added to the mixture and let to react overnight at 37.5 ^∘^C. The sample was divided in small aliquots and preserved at − 20 ^∘^C. Before analysis, 100 *μ* L of sample were diluted in 20 mM formic acid/ammonium formate buffer solution with pH 3.75.

### Mobile-phase preparation

Five aqueous (water) and organic (1:9 water/acetonitrile) mobile phases were prepared containing 20 mM formic acid/ammonium formate buffers with pH values 3.25, 3.50, 3.75, 4.00, and 4.25 respectively.

### Experimental design

The influence of column temperature and pH of the mobile phase on the retention time of each compound in the model sample was determined by means of experimental design. In order to determine possible non-linear effects with a good resolution, more than two typical experimental levels should be modeled, with the cost of extra number of experiments. Thus, a full factorial design with five levels for the two factors (pH of the mobile phase and column temperature) was generated, corresponding to a total of 25 chromatographic runs. In addition, the center point was replicated five times as controls and to access experimental errors due to other possible uncontrollable sources of variability between runs. The levels are represented in Table 1.

**Table 1:** Experimental values of the factors for the five-level full factorial design

Level	− 2	− 1	0	+ 1	+ 2
pH	3.25	3.50	3.75	4.00	4.25
Temperature (^∘^C)	35.0	37.5	40.0	42.5	45.0

### Data analysis

The data analysis and calculations (PCA and MLR) were performed in MATLAB 2017b (Mathworks, Natick, MA). TracMass 2, an open-source program running in MATLAB environment [10], was employed to extract the chromatographic ions (XIC) from the raw data and align the retention times across samples. The parameters used are represented in Table 2. Furthermore, in-house algorithms were employed to detect isotopes and adducts. These species were removed from the data, maintaining only the monoisotopic species, and a total of 98 species were considered in the calculations. These calculations, namely multi-linear regression (MLR), principal component analysis and regression (PCA and PCR), and partial least squares regression (PLS), were also preformed using also in-house MATLAB scripts. The ASCA algorithm for MATLAB was obtained from literature [24].

**Table 2:** TracMass 2 parameters

	Parameter	Value
Tracking	minLength	20
	minIntensity	30000
	mzTolerance	0.005
	mzTransformation	‘Square root’
Peak detection	ZAF Sigma 1	0.09
	ZAF Sigma 2	0.18
	gaussSigma	0.0048
	nSignalToNoise	20
	stdFiltWidth	6
Warping alignment	deltaTime	7
	deltaMass	0.01

## Results and discussion

### Experimental design regression coefficients

**Fig. 1:**
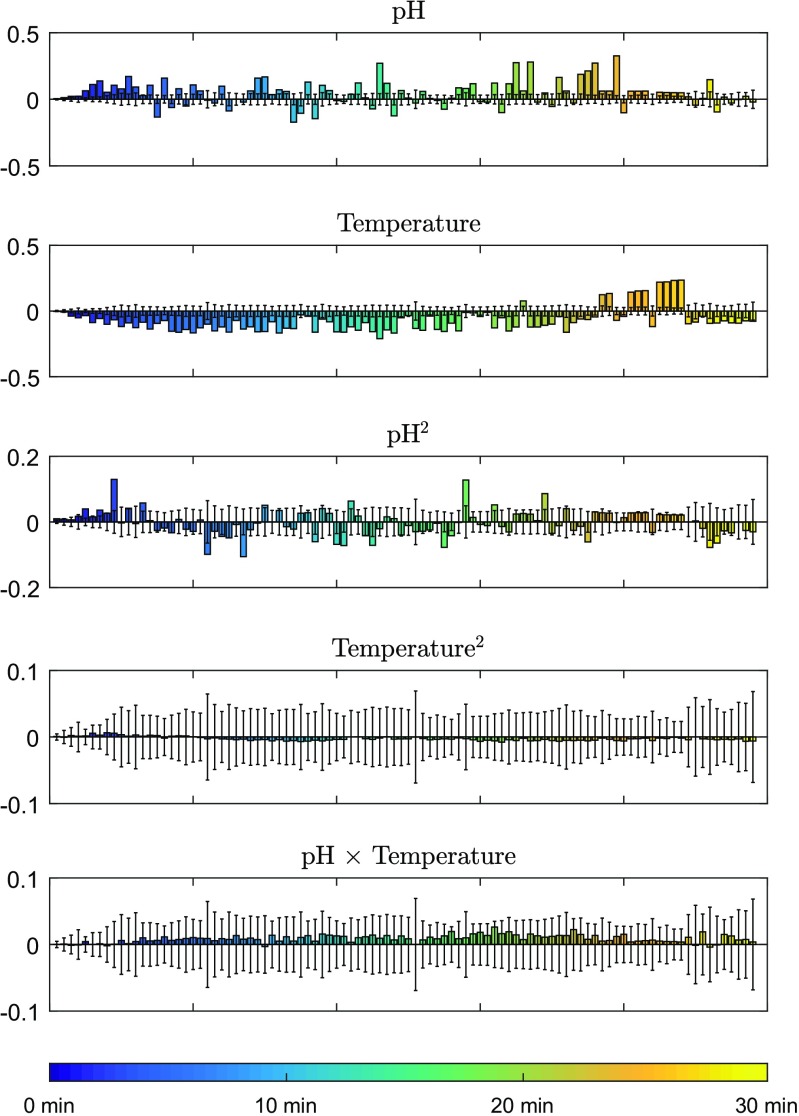
Factorial design MLR coefficients for 98 compounds (sorted according to their average elution times). The error bars represent confidence intervals with 95% confidence level, calculated from the retention times measured in five independent replicates analyzed in the center point conditions on different days

The experimental design MLR coefficients for the peaks of the 98 studied compounds, sorted according to their elution times, are represented in Fig. 1. The pH coefficients are both positive and negative, and their magnitudes vary from compound to compound, independently of the elution times. The temperature, on the other hand, exhibits mostly negative coefficients, which means that higher temperatures decrease retention times, except for ten compounds that display the opposite behavior. The quadratic term for the pH effect appears to be significant, whereas for the temperature, it does not. The coefficients corresponding to the interaction between pH and temperature have a positive effects. However, these coefficients also appear to be insignificant, as their magnitudes are lower than the errors due to repeatability (error bars), which were calculated as confidence intervals from five replicates in the center point performed in different days.

### PCA of the retention time data

**Fig. 2:**
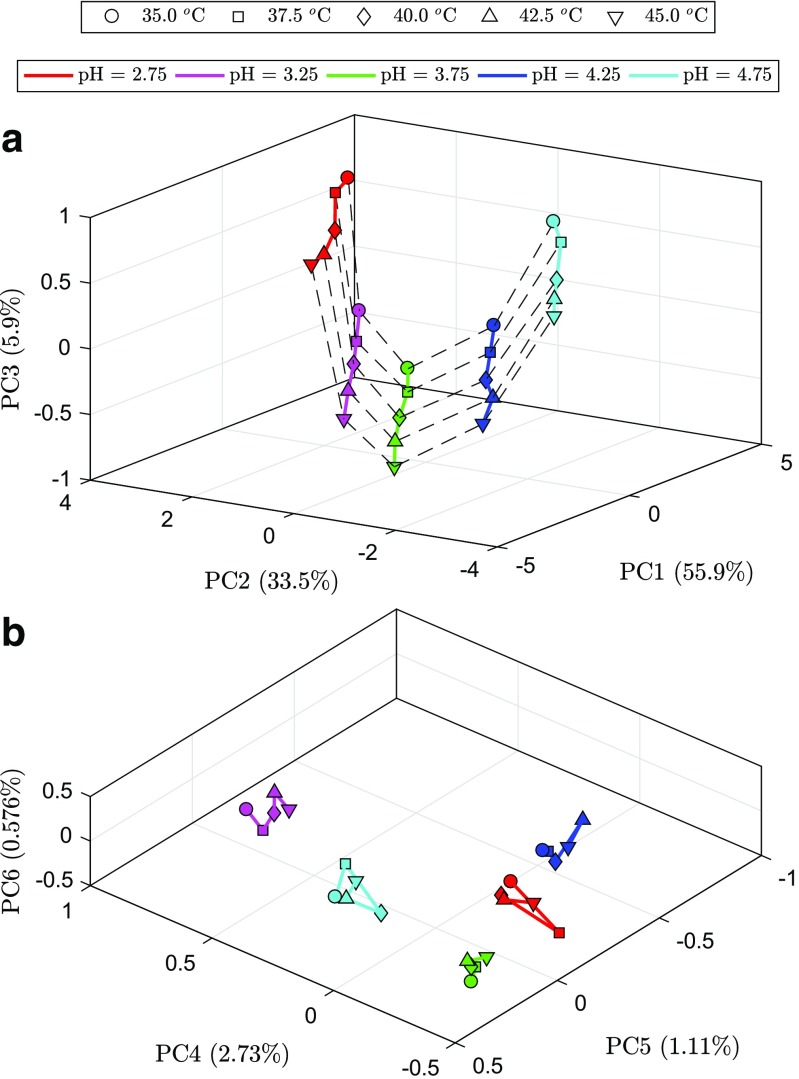
Principal component analysis of the experimental retention time data of 98 compounds in 25 runs. **a** First three principal components. **b** Fourth to sixth principal components

The PCA of the experimental responses, i.e., the retention times of the 98 selected compounds in 25 experimental design runs (Fig. 2), reveals a very well-defined structure. From the observation of this figure, it is noticeable that the temperature has linear trends, whereas the pH exhibits a curvature, which suggests a quadratic effect. This result demonstrates a qualitative agreement between PCA scores, **T** and the design matrix, **D**. The ASCA scores also confirm this, as represented in Figs. S3, S4, and S5 in the Electronic Supplementary Material (ESM).

### PCR of the retention time data

**Fig. 3:**
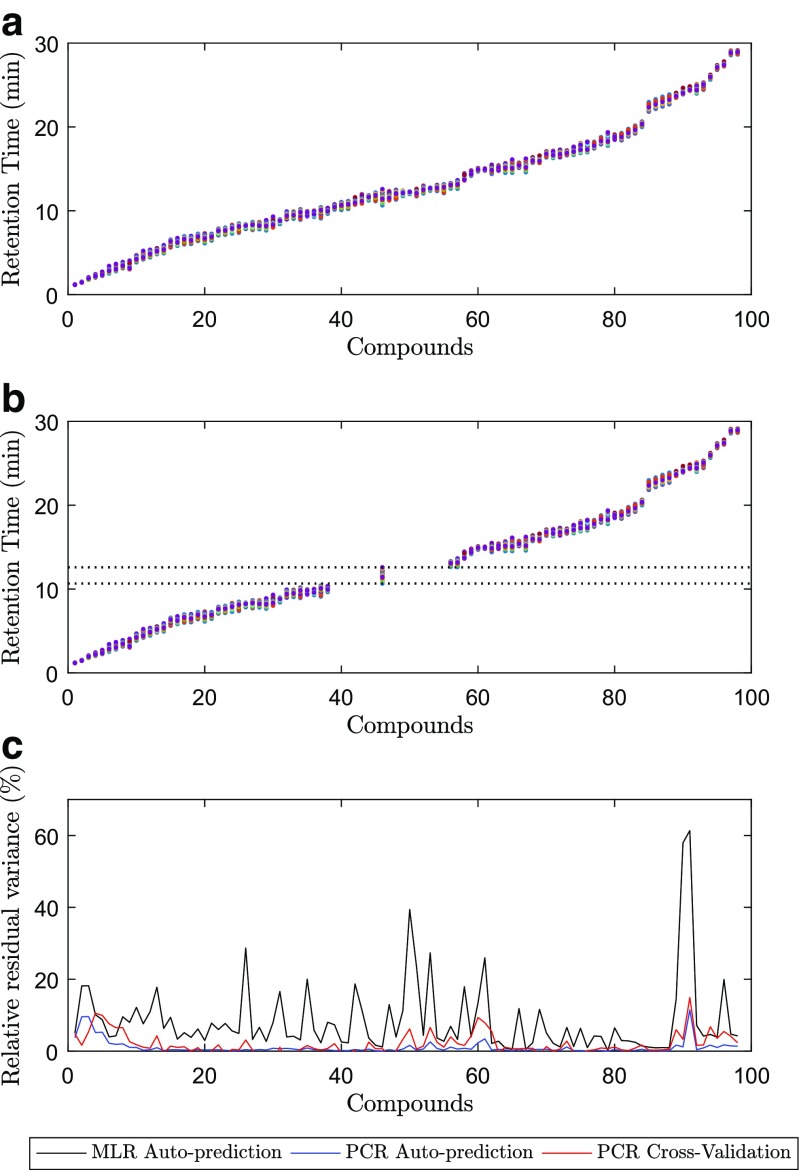
Quality in prediction by MLR and PCR. **a** Retention time profiles for 98 compounds (sorted according to their elution times). **b** Example used in a PCR model for one compound. Between the red lines is the predictor vector and outside is the calibrant data. **c** Relative residual variance of auto-prediction and cross-validation for the PCR models and auto-prediction for the MLR models

By means of principal component regression (PCR), it is possible to create calibration models to predict the retention times of each compound while calibrating with the data from the remaining compounds. To obtain more trustworthy models, these calibration blocks were built in such way that no peaks between the calibrant data and predictor vector overlapped, as illustrated in Fig. 3a, b. This demonstrates that all obtained retention time data are correlated, i.e., the retention time shifts of each individual compound are correlated with those of other compounds in the mixture when varying the chromatographic conditions (in this case, the pH of the mobile phase and the column temperature). Figure 3b represents an example on how one of the 98 calibration models was created, i.e., all the vectors outside the lines are the calibration predictors (*X*), and inside the lines is the response vector (*y*). The removal of vectors containing overlapping data with the response vector was performed in order to prove that the models are built on information from other analyses and that the X-block does not contain isotopes, adducts, or fragments that would make the modeling trivial. Using the MLR experimental design coefficients in auto-prediction and calculating the relative residual variance (Fig. 3c), it is noticeable that the quality of the models is not as good as with PCR. This lack of fit suggests that along with the effects that were studied, i.e., linear and quadratic pH and linear temperature effects, there may be other uncontrollable factors that were not included in the model, or errors associated with the nominal parameters, i.e., the column temperature and the pH of the mobile phase values are prone to deviate from those established by the experimental design levels. With PCR and with a correct choice of principal components (PCs), the predictions were better than with those of MLR. In this study, six PCs were used to create the PCR models because, as observed in Fig. 2, there appears to be structure up until the sixth PC. The total PCA variance explained is 99.7%. Also, in the predictions of the design levels, as described bellow (Fig. 5), the RMSE of calibration and cross-validation suggests that a better PCR model is obtained with six principal components. Cross-validation is a typical approach in PCR and partial least squares (PLS) to determine the best number of latent components to include in a model [23]. The PCR cross-validation RMSE results indicate that not much over-fitting occurred when modeling with this number of PCs. Moreover, the non-linearity associated with the pH of the mobile phase also adds at least one extra principal component. Uncontrollable factors are implicitly included in a PCR model, whereas in MLR, these have to be explicitly included in the regression model equation. These results are practically the same when modeled by means of PLS (Figs. S1 and S2 in ESM) and comply with the theory behind the development of the GFHT algorithm [8–10], where the shifts of peaks with unknown correspondence can be predicted from calibration models of the shifts that have known correspondence because shifts have patterns that can be attributable to a finite number of causes related to chromatographic instability. Here, it has been demonstrated that there is at least as much information about the column temperature and the pH of the mobile phase in the shifts of the peaks as there is in the design matrix. Therefore, the GFHT approach to alignment is sound in the sense that peak shifts can be trusted to reliably predict the position of missing or ambiguous peaks in other clusters or groups of peaks belonging to the same analyte.

### Experimental design coefficients by MLR and PCA

**Fig. 4:**
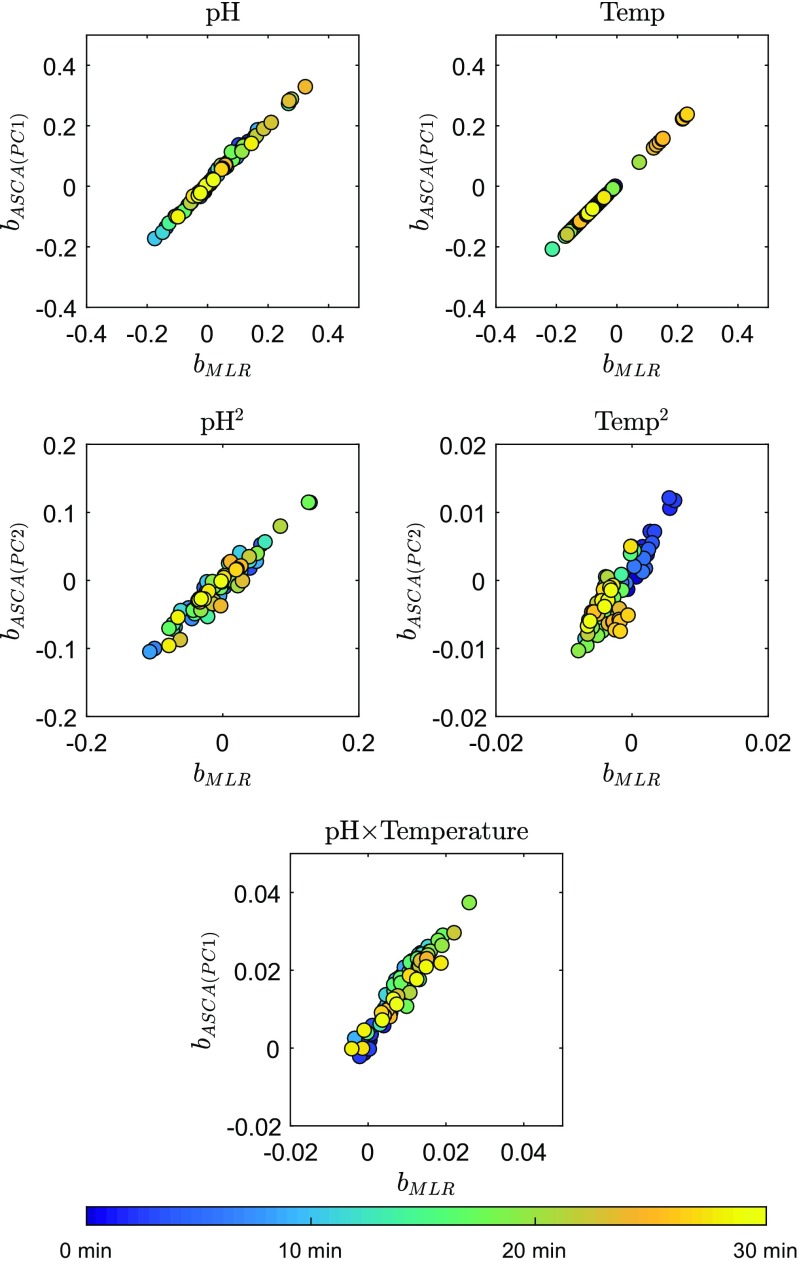
Regression coefficients calculated by ASCA against MLR. The colors represent the elution times of the compounds. Closely related compounds or compounds that are affected by temperature and pH to the same extent will cluster together from having about the same coefficients both when fitting the retention time shifts with MLR and with PCR

**Fig. 5:**
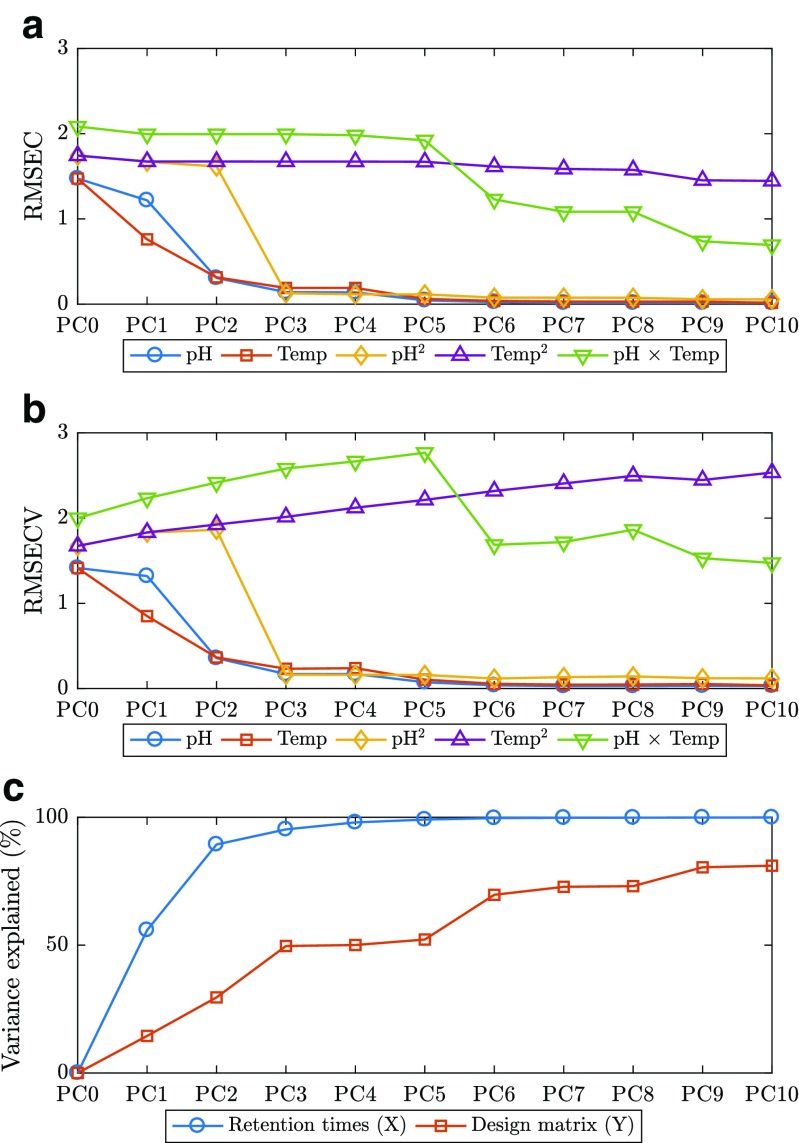
Quality in prediction of the experimental design levels by PCR using different numbers of principal components. Root mean square errors of calibration (**a**) and cross-validation (**b**). The variance explained **c** in the PCR auto-prediction of the experimental design levels from the retention times

When comparing the DoE coefficients obtained from MLR and from the ASCA loadings (Fig. 4), an agreement between the results is noticeable for all considered factors and interaction. However, the column temperature quadratic and interaction between pH of the mobile phase and column temperature terms have very small coefficients and it is expected that modeling these effects may be greatly affected by noise. This is confirmed when modeling the experimental design levels from the retention time data, where the terms with small coefficients result in poor auto-prediction and cross-validation (Fig. 5). When calculating the coefficients from the PCA loadings (instead of ASCA) for these two factors, they do not match those obtained with MLR (Fig. S6 in ESM). As explained above, other uncontrollable factors besides column temperature and pH of the mobile phase, and possible deviations from the experimental design nominal values (levels) of these factors, are modeled by PCA but not included in a MLR model. Nonetheless, these contributions are very small in comparison to the studied imposed factors in these performed experiments.

## Conclusions

An application of experimental design to study the influence of column temperature and pH of the mobile phase on the chromatographic separation of peptides revealed well-defined retention time shift patterns. The temperature affects retention times in a linear fashion, which means that peaks will shift linearly with changes in the column temperature. On the other hand, the pH of the mobile phase affects the retention times in a quadratic fashion. Yet, different peaks were affected to different extents and six patterns of peak shifts were found varying these two factors. Moreover, it was demonstrated that the retention time can be modeled better from the retention times of other compounds by means of PCR than from the experimental design. This provides experimental evidence that supports the previously reported generalized fuzzy Hough transform alignment algorithm, which aligns shifted peaks based on patterns derived from the data, by demonstrating that the GFHT approach to alignment is trustworthy, in the sense that shifts of peaks from other analytes can reliably predict the position of missing or ambiguous peaks from groups of peaks belonging to the same analyte. In all, a better understanding of the shift patterns may contribute to the development of better alignment algorithms.

## Electronic supplementary material

Below is the link to the electronic supplementary material.

(PDF 648 KB)
