# Supplementary material for: Elucidation of chromatographic peak shifts in complex samples using a chemometrical approach
Source: Anal Bioanal Chem. 2018 Jun 14;410(21):5229–35. doi: 10.1007/s00216-018-1173-9 (PMC6061714; doi:10.1007/s00216-018-1173-9)
Supplement: Supplementary file 1 — (PDF 648 KB) [file 216_2018_1173_MOESM1_ESM.pdf]

Analytical and Bioanalytical Chemistry

Electronic Supplementary Material

Elucidation of chromatographic peak shifts in complex samples using a chemometrical approach

Pedro F.M. Sousa, Angela de Waard, K. Magnus Åberg

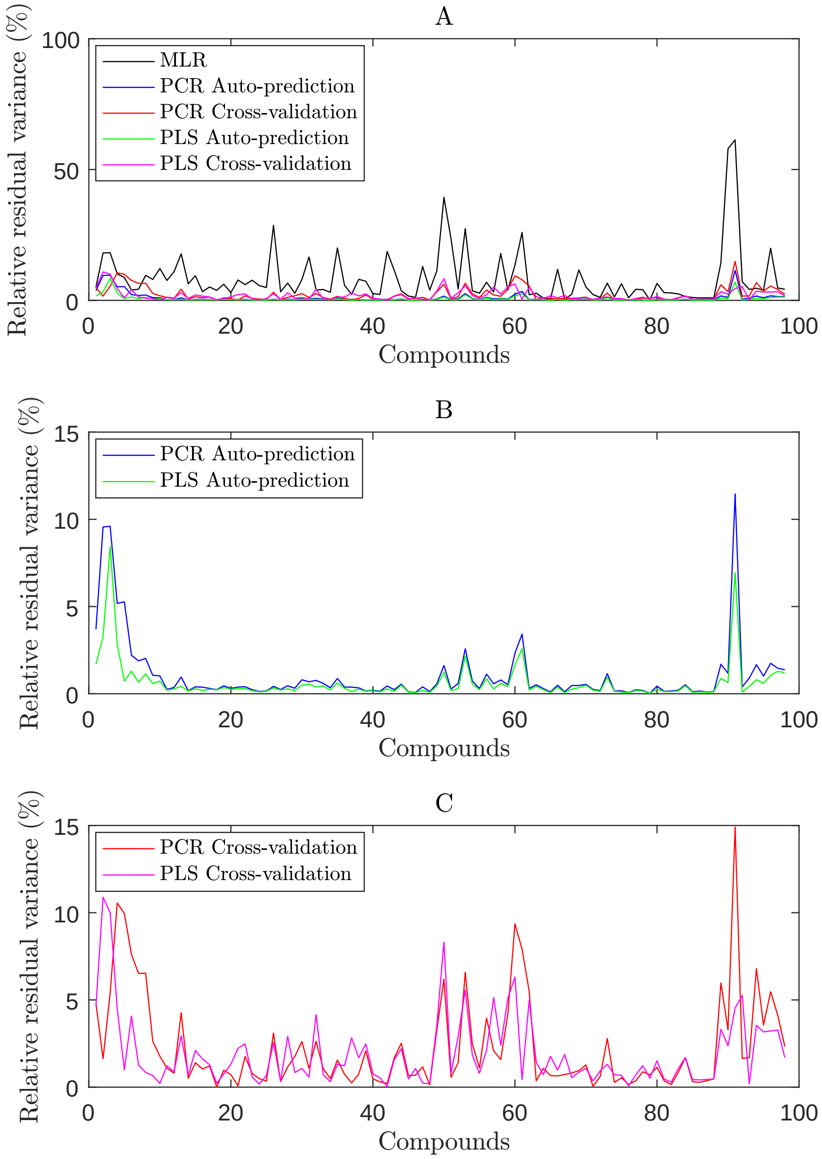

Fig. S1 Quality in prediction of retention times by MLR and PCR and PLS. (A) Relative residual variance of auto-prediction and cross-validation for the PCR and PLS models and auto-prediction for the MLR models. (B) Auto-prediction for PLS and PCR. (C) Cross-validation for PLS and PCR

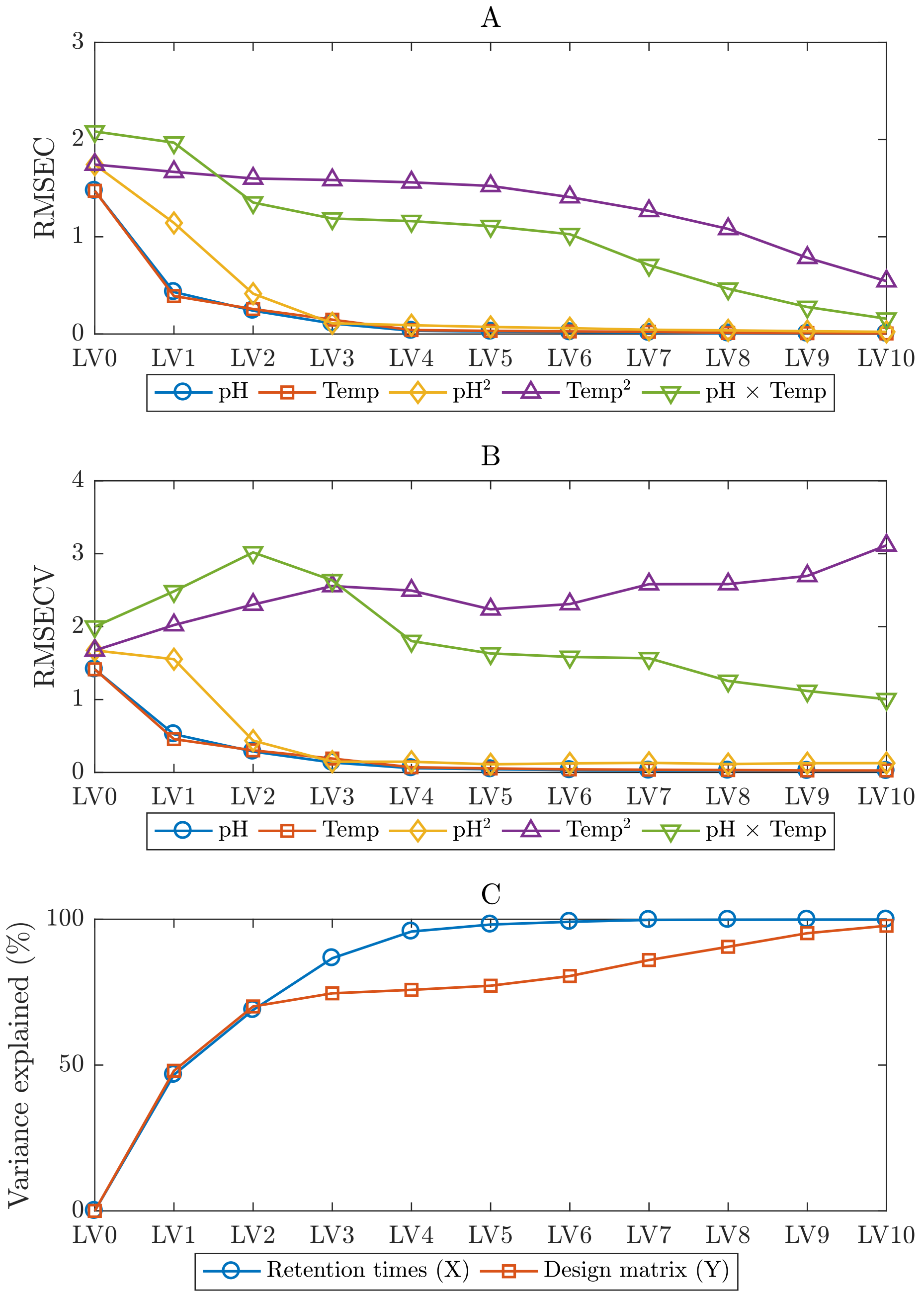

Fig. S2 Quality in prediction of the experimental design levels by PLS using different number of latent variables. Root mean square errors of Calibration (A) and Cross-validation (B). The mean variance explained (C) by the PLS models for the calibration of the five parameters

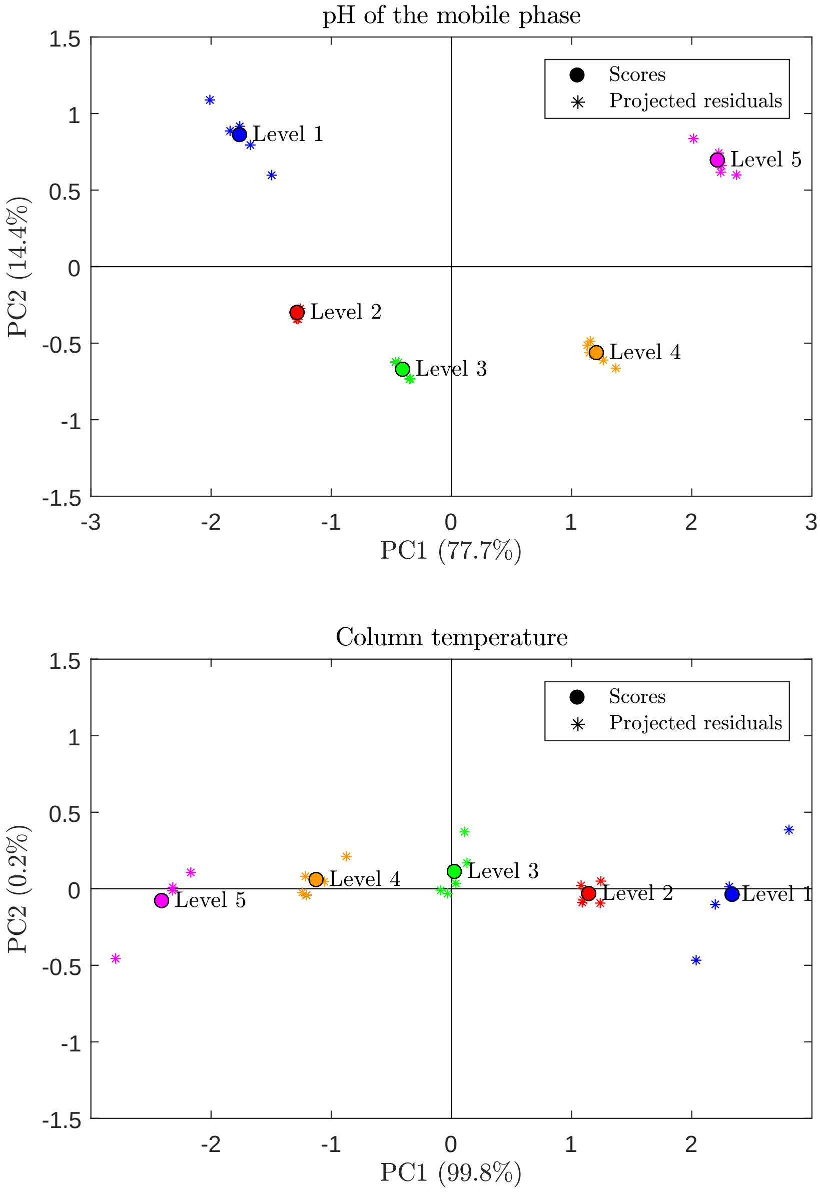

Fig. S3 ASCA scores of the studied factors and projected residuals. The pH of the mobile phase exhibits a curved shape plot, which confirms that this factor has a quadratic relationship with retention time shifts. The column temperature has a well defined linear trend

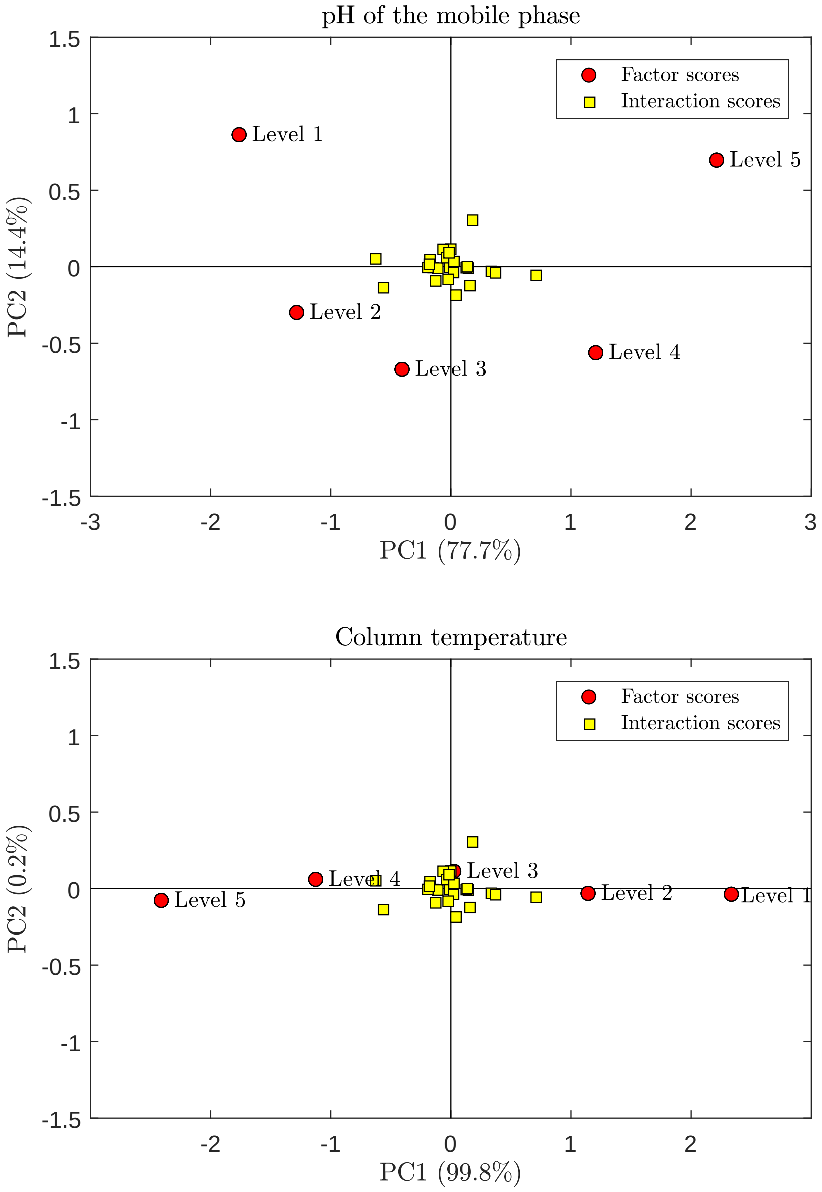

Fig. S4 ASCA scores of the pH of the mobile phase and column temperature levels and interation scores. The interaction scores are much less significant than the factors' scores

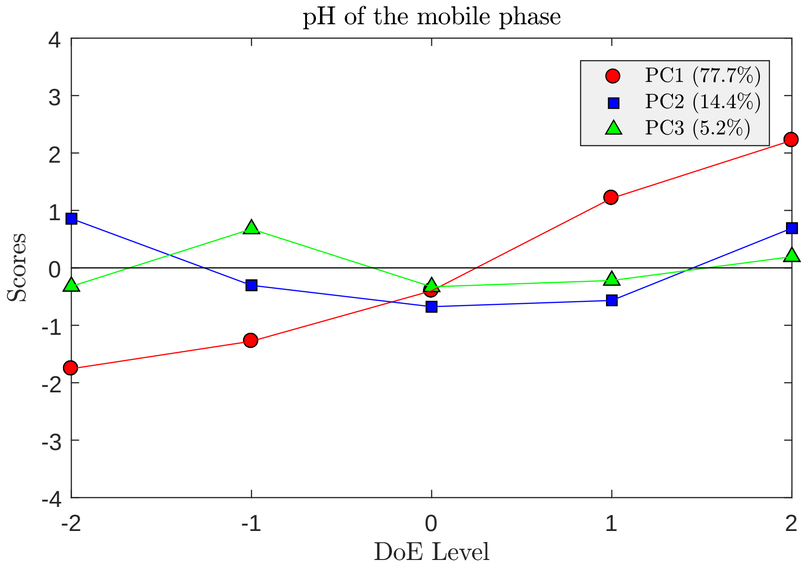

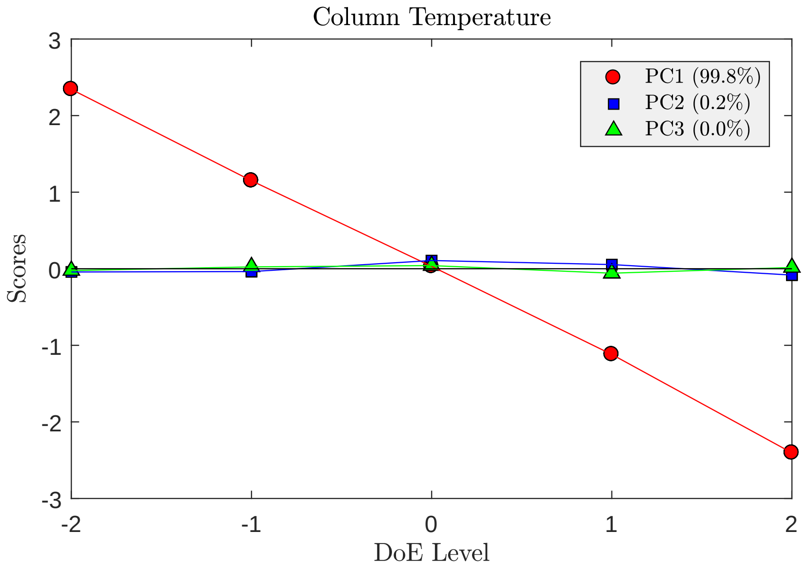

Fig. S5 ASCA scores of the studied factors for different principal components. The pH of the mobile phase has a close-to-linear reletionship between the scores and the level for PC1, PC2 exhibits a curvature, and PC3 has scores close to zero except for level -1. This confirms that this factor has a quadratic relationship with retention time shifts. The column temperature has a well defined linear trend

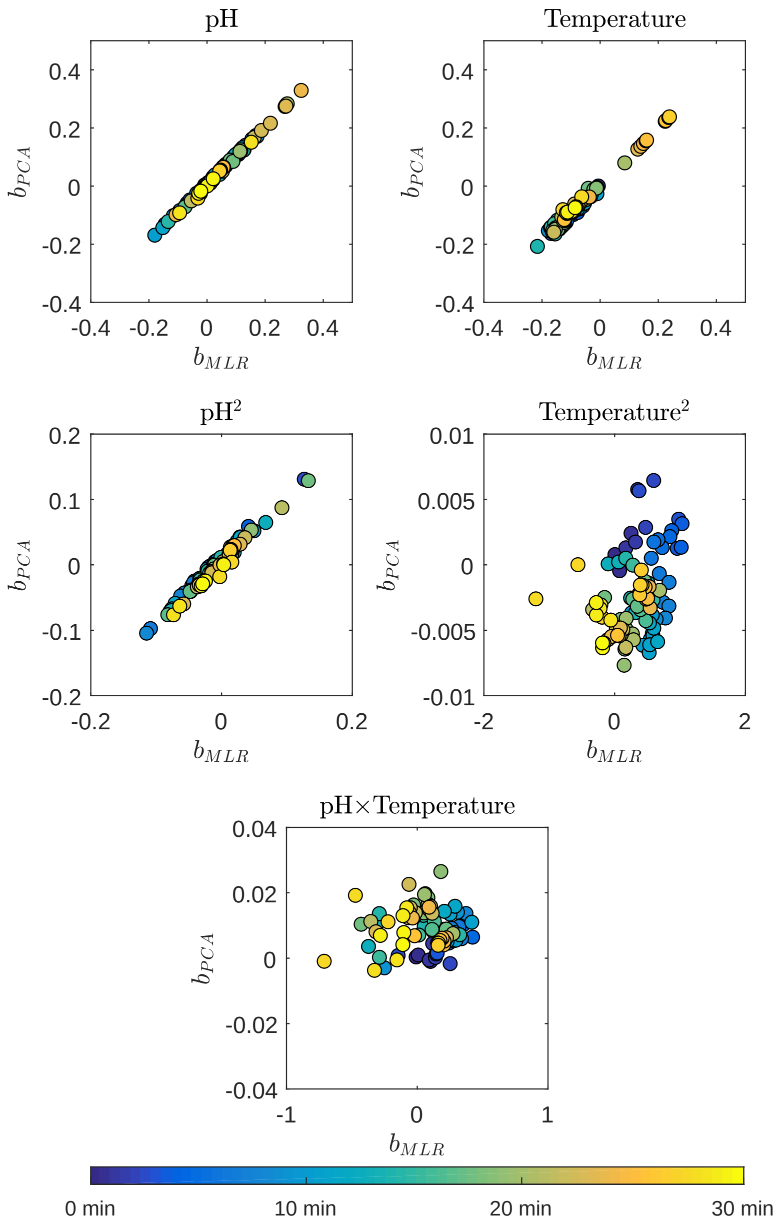

Fig. S6 Regression Coefficients calculated from PCA loadings and calculated by MLR. The colors represent the elution times of the coumpounds. Closely related compounds or compounds that are affected by temperature and pH to the same extent will cluster together from having about the same coefficients. The squared Temperature and interaction effects do not match aswhen the DoE coefficients are calculated from the ASCA loadings
